# The Serotonin Receptor Subtype 5b Specifically Interacts with Serotonin Receptor Subtype 1A

**DOI:** 10.3389/fnmol.2017.00299

**Published:** 2017-09-21

**Authors:** Sabine Niebert, Gijsbert J. van Belle, Steffen Vogelgesang, Till Manzke, Marcus Niebert

**Affiliations:** ^1^Department of Maxillofacial Surgery, University Medical Center Göttingen, Germany; ^2^Institute of Cardiovascular Physiology, University Medical Center Göttingen, Germany; ^3^Institute of Neuro- and Sensory Physiology, University Medical Center Göttingen, Germany; ^4^Center Nanoscale Microscopy and Molecular Physiology of the Brain (CNMPB), University Medical Center Göttingen, Germany

**Keywords:** 5-ht5b receptor, 5-HT1A receptor, Rett syndrome, signaling, cAMP

## Abstract

Previously, we described the dysregulation of serotonin (5-HT) receptor subtype 5b (5-ht_5b_) in a mouse model of Rett syndrome (RTT). 5-ht_5b_ has not been extensively studied, so we set out to characterize it in more detail. Unlike common cell surface receptors, 5-ht_5b_ displays no membrane expression, while receptor clusters are located in endosomes. This unusual subcellular localization is at least in part controlled by glycosylation of the N-terminus, with 5-ht_5b_ possessing fewer glycosylation sites than related receptors. We analyzed whether the localization to endosomes has any functional relevance and found that 5-ht_5b_ receptors can specifically interact with 5-HT_1A_ receptors and retain them in endosomal compartments. This interaction reduces 5-HT_1A_ surface expression and is mediated by interactions between the fourth and fifth trans-membrane domain (TMD). This possibly represents a mechanism by which 5-ht_5b_ receptors regulate the activity of other 5-HT receptor.

## Introduction

G-protein coupled receptors (GPCRs) make up ~2% of all cellular proteins and constitute one of the most important pharmaceutical drug targets. With all their diversity, GPCRs share some defining features, e.g., they are composed of seven trans-membrane domains (TMD) with three extracellular and four intracellular loops, and many receptors need to form homo- or heterodimers to become functional active (Gurevich and Gurevich, 2008; Schwenk et al., 2010; Pellissier et al., 2011). The receptors relate extracellular signals by activating G-proteins acting on either one of the main effectors calcium (G_q_ via Phospholipase C) or cyclic adenosine monophosphate (cAMP). cAMP can be up- or down-regulated via stimulatory (G_s_) or inhibitory (G_i_/G_0_) G-proteins who activate or inhibit the adenylate cyclase, respectively. Increases in cAMP may directly activate cyclic nucleotide gated (CNG) ion channels (Kaupp and Seifert, 2002) or protein kinase A (PKA; Meinkoth et al., 1993). Intracellular sensors for cAMP like exchange protein activated by cAMP (EPAC; Bos, 2006) or popeye domain-containing proteins (Popdc, Simrick et al., 2013) function through small GTPases.

Serotonin (5-HT) is a widespread neurotransmitter in the nervous systems, with numerous functions in sensory-motor, autonomic and behavioral systems (Azmitia, 2001). It is also a hormone and paracrine factor in non-neural tissues (Matsuda et al., 2004; Slominski et al., 2005). In mice, 5-HT receptors comprise seven groups with multiple subtypes (5-HT_1A/B/D/F_, 5-HT_2A/B/C_, 5-HT_4_, 5-ht_5a/b_, 5-HT_6_ and 5-HT_7_; Hoyer et al., 2002) for a total of 12 GPCRs, while 5-HT_3A/B_ acts as an ion channel. In humans, four additional 5-HT receptors exist that do not have a known ortholog in mice: 5-HT_1E_ is another GPCR, while 5-HT_3C/D/E_ form ion channels.

While many 5-HT receptors have been extensively studied (Niebert et al., 2011; Pellissier et al., 2011; Renner et al., 2012), our knowledge of 5-ht_5_ receptors is rather limited. Rodents have been shown to possess two functional 5-ht_5_ receptor subtypes, 5-ht_5a_ (Plassat et al., 1992) and 5-ht_5b_ (Matthes et al., 1993), with their expression being restricted to the brain (Rees et al., 1994). The existence of both receptors was confirmed for humans (Rees et al., 1994; Grailhe et al., 2001; Nelson, 2004), which possess a functional gene for 5-ht_5a_, while the coding sequence of the 5-ht_5b_ subtype is interrupted by several stop codons and insertional mutations, making the gene apparently non-functional.

5-ht_5b_ is expressed in many regions throughout the murine brain like bulbus olfactorius, hippocampus, or cerebellum at low levels. However, 5-ht_5b_ was found to be upregulated under the control of ATF-7 in mice undergoing social stress (Maekawa et al., 2010). Also, 5-ht_5b_ dysregulation was described in mouse models of Rett syndrome (RTT), a severe neurodevelopmental disorder caused by mutations in the transcription factor MeCP2. We found 5-ht_5b_ to be developmentally regulated in the brainstem of *Mecp2^−/y^* mice, where it showed 75-fold upregulation (Vogelgesang et al., 2017), while 5-ht_5b_ was also increased in astrocytes of *Mecp2*^308/y^ mice (Delépine et al., 2015). We showed that a full length as well as a truncated protein of 5-ht_5b_ can be detected by Western blot in murine brain lysates (Vogelgesang et al., 2017). Truncation of receptors has been described many times, but previously identified truncated GPCRs were predominantly retained in the endoplasmic reticulum (ER; Karpa et al., 2000; Leung et al., 2007; Gonzalez et al., 2011).

5-ht_5b_’s massive dysregulation in a disease model for RTT prompted us to more closely investigate the reasons for and the physiological significance of its subcellular localization.

## Materials and Methods

### Ethical Statement

The experimental procedures were performed in accordance with European Community (EU Directive 2010/63/EU for animal experiments) and National Institutes of Health guidelines for the care and use of laboratory animals. The study was approved by the Georg-August-University, Göttingen and the approval ID T12/18 was assigned to this work.

### Animals

C57BL/6J mice were kept in a temperature- and humidity-controlled 12 h light-dark cycle and had free access to water and standardized pellet food. The *Mecp2* knockout mouse (*Mecp2*^−*/y*^), strain B6.129P2(C)-Mecp2^tm1-1Bird^ (Guy et al., 2001) obtained from The Jackson Laboratory (Bar Harbor, ME, USA) was maintained on a C57BL/6J background. For this work we used wild-type and hemizygous *Mecp2*^+/−^ females.

### Cell Culture, Transfection and Plasmids

Murine neuroblastoma cell line N1E-115 was obtained from the American Type Culture collection (ATCC). Cells were grown in Dulbecco’s modified Eagle’s medium (DMEM) containing 10% fetal calf serum (FCS) and 1% penicillin/streptomycin at 37°C under 5% CO_2_.

For transient transfection, cells were seeded in cell culture dishes and transfected with indicated plasmids using Lipofectamine2000 Reagent (Invitrogen) according to the manufacturer’s instruction.

Primary neurons were obtained from dissociated hippocampi as detailed in Revelo et al. (2014). In brief, hippocampi were extracted from 1 day-old animals. They were incubated for 1 h in enzyme solution (10 ml DMEM, 2 mg cysteine, 100 mM CaCl_2_, 50 mM EDTA and 25 U papain, equilibrated with carbogen for 10 min, and sterile filtered). Before mechanical dissociation, cells were washed thoroughly with HBSS (Invitrogen, Waltham, MA, USA) and were incubated for 15 min in inactivating solution (2 mg albumin and 2 mg trypsin inhibitor in 10 ml FCS-containing DMEM medium). Sterilized coverslips were coated overnight with 1 mg/ml poly-L-lysine and were incubated with plating medium (MEM supplemented with 10% horse serum, 3.3 mM glucose and 2 mM glutamine). Neurons were plated at a concentration of ~30,000/cm^2^ and were left to adhere for 1–4 h. After adhesion, the medium was changed to Neurobasal-A medium (Gibco, Life Technologies, Carlsbad, CA, USA) containing 1:50 B27 supplement (Gibco) and 1:100 GlutaMAX (Gibco). Neurons were kept in culture at 37°C and 5% CO_2_.

Expression constructs for 5-HT receptors 5-HT_1A_ and 5-HT_7_ with either immuno- (HA/myc) or fluorescent tags have been described previously (Renner et al., 2012), while 5-HT receptors 5-HT_2C_, 5-HT_4_, 5-ht_5a_, 5-ht_5b_ and 5-HT_6_ were generated from murine corresponding deoxyribonucleic acid (cDNA). Brain tissue was explanted and used for total ribonucleic acid (RNA) isolation with the OLS RNA kit (OLS, Germany) according to the manufacturer’s instructions. The total RNA was used in one-step RT-PCR (Invitrogen) and resulting PCR fragment was cloned into pTarget expression vector (Promega). Primer sequences are given in Table 1. To obtain a C-terminal fluorophore fusion construct, the receptor and the fluorophores CFP, GFP, YFP or mRFP/mCherry were amplified individually, and fusion PCR was used to combine the cDNAs. The resulting PCR fragment was cloned into the pTarget expression vector (Promega). To test the hypothesis of a truncated 5-ht_5b_ receptor, we amplified the unlabeled and the mCherry fusion construct and cloned the fragments into pTarget. The truncated 5-ht_5b_ comprises the nucleotides from position 520 to 1113. As a control, a truncated 5-HT_7_ was prepared in the same way. The truncated 5-HT_7_ comprises the nucleotides from position 520 to 1347. Correct sequences of all constructs were determined by sequencing.

**Table 1:** List of primers used in this study for cloning.

*Htr1a-N10S*	for	CTTGGCCAGGGCA**G**CAACACCACAACG	NM_008308.4
	rev	CGTTGTGGTGTTG**C**TGCCCTGGCCAAG	
*Htr1a-N11S*	for	GGCCAGGGCAACA**G**CACCACAACGTCC
	rev	GGACGTTGTGGTG**C**TGTTGCCCTGGCC	
*Htr1a-N24S*	for	GGGACAGGCGGCA**G**CGATACTGGCCTC
	rev	GAGGCCAGTATCG**C**TGCCGCCTGTCCC	
*Htr1a-N30S*	for	ACTGGCCTCTCCA**G**CGTGACCTTCAGC
	rev	GCTGAAGGTCACG**C**TGGAGAGGCCAGT	
*Htr2c*	for	ATGGTGAACCTGGGCACTGCGG	NM_008312.4
	rev	TTACACACTACTAATCCTCTCGC	
*Htr4*	for	ATGGACAAACTTGATGCTAATG	NM_008313.4
	rev	TTAAGTATCACTGGGCTGAGC	
*Htr5a-FL*	for	ATGGATCTGCCTGTAAACTTG	NM_008314.2
	rev	TCATTGTTGCTTGGAGAAGAAG	
*Htr5b-FL*	for	ATGGAAGTTTCTAACCTCTC	NM_010483.3
	rev	TTATCTCTGCTTAGTAAAGAG	
*Htr5b-trunc*	for	ATGATCGCGATCACCTGGG	
	rev	TTATCTCTGCTTAGTAAAGAG	
*Htr6*	for	ATGGTTCCAGAGCCCGGCCC	NM_021358.2
	rev	TCAGTTCATGGGGGAACCAAG	
*Htr7-trunc*	for	ATGACCCTGTGCGTGATCAGC	NM_008315.2
	rev	TCATGTATCATGACCTTTTTTCCC	
*HRV-12*	5b-for	ATGGAAGTTTCTAACCTCTC	*5b(1−165) + 1A(88−1266)*
	fusion for	TGCTTTCACCGTGCTTGTGAACGTGACCTTCAGCTACCAAGTG	
	fusion rev	CACTTGGTAGCTGAAGGTCACGTTCACAAGCACGGTGAAAGCA	
	1a-rev	GCGGCAGAACTTGCACTTGATGATC	
*HRV-13*	5a-for	ATGGATCTGCCTGTAAACTTG	*5a(1−85) + 5b(139−1113)*
	fusion for	CTGCGCCCTAGTCGGCCTCCCTTCTCTGCTTTC	
	fusion rev	GAAAGCAGAGAAGGGAGGCCGACTAGGGCGCAG	
	5b-rev	TCTCTGCTTAGTAAAGAGGC	
*HRV-14*	1A-for	ATGGATATGTTCAGTCTTGGCC	*1a(1−102) + 5b(148−1113)*
	fusion for	CGTGACCTTCAGCGCTTTCACCGTGC	
	fusion rev	GCACGGTGAAAGCGCTGAAGGTCACG	
	5b-rev	TCTCTGCTTAGTAAAGAGGC	
*HRV-15*	5b-for	ATGGAAGTTTCTAACCTCTC	*5b(1−1044) + 1A(1183−1266)*
	fusion for	CGTTCTTCAACCCCCTCAACCCAGTTA	
	fusion rev	TAACTGGGTTGAGGGGGTTGAAGAACG	
	1A-rev	GCGGCAGAACTTGCACTTGATGATC	
*HRV-17*	1A-for	ATGGATATGTTCAGTCTTGGCC	*1A(1−669) + 5b(673−1113)*
	fusion for	CAGAGCCGCGCGCCCTCTAGCGGTGGTGCTC	
	fusion rev	GAGCACCACCGCTAGAGGGCGCGCGGCTCTG	
	5b-rev	TCTCTGCTTAGTAAAGAGGC	
*HRV-18*	1A-for	ATGGATATGTTCAGTCTTGGCC	*1A(1−456) + 5b(526−1113)*
	fusion for	GAGGACGCCCCGGCGCGCGATCACCTGGGCAC	
	fusion rev	GTGCCCAGGTGATCGCGCGCCGGGGCGTCCTC	
	5b-rev	TCTCTGCTTAGTAAAGAGGC	

*Accession numbers are given for the murine gene used to generate the primers*.

### Generation of Glycosylation-Deficient 5-HT_1A_ Constructs

Wild type 5-HT_1A_ can be glycosylated at four positions (N10, N11, N24, N30) at the N-terminus. Following the glycosylation experiments on 5-ht_5a_, we used site-directed mutagenesis (Quickchange II, Agilent) to change each individual Asparagine to Serine (Dutton et al., 2008). To generate clones with multiple exchanges, we performed subsequent rounds of mutagenesis. Primers used for mutagenesis are given in Table 1 and correct sequences of all constructs were determined by sequencing.

### Generation of Hybrid Receptors

The modular structure of the GPCRs facilitates easy exchange of domains between receptors, which has previously been used successfully to generate versions with completely new characteristics (Conklin et al., 2008). 5-HT receptors are composed of seven TMD, which are connected by three extra- (ECL) and three intra-cellular loops (ICL). The extra-cellular N-terminus takes part in ligand attraction, while the intra-cellular C-terminus often, but not always features a PDZ-like domain and takes part in membrane anchoring or protein binding. The problem with this approach is the difficulty to predict domain boundaries, as incorrectly chosen domains may prevent correct folding and therefore render the new hybrid receptor functionless. As we employed this strategy multiple times, we took care to validate the correct function of the new hybrid receptors. Our first quality check was their production of a distinct staining, be it intracellular or membrane bound. All variants that showed a diffuse staining were assumed to be incorrectly folded and were not used for further studies. As long as the receptor showed at least some membrane localization, we also tested the hybrid receptor for its reaction to its natural ligand 5-HT.

DNA sequences coding for TMDs were identified using TMHMM server 2.0 (Krogh et al., 2001). Hybrid receptors were generated using fusion PCR. Therefore, the DNA sequences to be fused were separately amplified by PCR using specific primers, introducing a small overlapping DNA region. After purification of both PCR products by gel extraction, 10 ng of each DNA was used as template for a subsequent fusion PCR using primers specific for the full-length hybrid open reading frame. The primers used for generation of different variants of *Htr1a*-*Htr5b* hybrid receptor variants are given in Table 1.

### RT-PCR

The total RNA of homogenized tissue from specific brain regions was isolated using the Trizol^®^ method according to manufacturer’s instructions (GibcoBRL) and its concentration was determined using the nanodrop ND-1000 spectrophotometer followed by its quality and integrity measurement by electrophoresis on RNA 6000 LabChip^®^ kit (Agilent 2100 Bioanalyzer). The RNA was transcribed into the cDNA using the iScript cDNA Synthesis Kit (BioRad). The primer pairs used for RT-PCR are the same used previously for quantitative RT-PCR (Vogelgesang et al., 2017).

### Immuno-Staining Procedures

To obtain tissue, mice (P40) were deeply anesthetized with isoflurane (1-Chloro-2,2,2-trifluoroethyl-difluoro-methylether, Abbott, Germany) until they were unresponsive to pain stimuli. A thoracotomy was performed and animals were transcardially perfused with 50 ml of 0.9% NaCl followed by 200 ml of 4% phosphate-buffered formaldehyde (10 ml/min). The brain was removed and post-fixed for 4 h with the same fixative at 4°C. Whole brains were stored in 1% formaldehyde in PBS at 4°C. Before sectioning, brains were equilibrated in HEPES buffer (7, 5 g NaCl, 0.3 g KCl, 0.06 g KH_2_PO_4_, 0.13 g Na_2_HPO_4_, 2 g Glucose, 2.4 ml 10 mM HEPES, 0.1 g MgCl_2_, 0.05g MgSO_4_, 0.165 g CaCl_2_, pH 7.4) for 48 h, cryoprotected in 15% sucrose in PBS for 24 h followed by equilibration in 30% sucrose in PBS for 24 h at 4°C, and then frozen at −80°C. Series of 30 μm thick brain sections were cut using a freezing microtome (Frigocut, Reichert-Jung, Germany). Sections were stored in HEPES buffer. All buffers were supplemented with a small amount of sodium azide.

The polyclonal antibody against the murine 5-ht_5b_ receptor was generated by immunizing rabbits with a 15mer peptide representing the C-terminus of the receptor (NP_034613.1; NH2-KNYNNAFKSLFTKQR-COOH). Peptides were coupled to keyhole limpet hemocyanin (KLH) and 300 μg KLH-coupled peptide in Hunter’s adjuvant (TiterMax Gold, Sigma) was administered five times (28-days-intervall). Antibodies were purified on an antigen-coupled CNBr-activated Sepharose^®^ 4B column. The eluate was dialyzed against two changes of 5 l PBS for 24 h at 4°C, and finally concentrated to at least 1 μg IgG/μl.

Primary antibodies were diluted 1:100 in blocking buffer (PBS, 0.1% Triton-X100, 1% Tryptone/Peptone) and incubated for 60 min at RT. After washing in washing buffer (PBS, 0.05% Tween20, 0.3% Triton X100), sections were incubated for 1 h at RT in the dark with anti-rabbit atto647-conjugated secondary antibodies (Sigma-Aldrich, Cat. No. 40839) diluted 1:400 in blocking buffer. Sections were mounted onto microscope-slides and cover-slipped with Mowiol.

To analyze interaction of HA-5-HT_1A_ and 5-ht_5b_mCherry in N1E-115 neuroblastoma cells, double-transfected live cells were first stained at low temperatures with rabbit-anti-HA-tag primary antibodies for 5 min and anti-rabbit atto647-conjugated secondary antibodies for 5 min to avoid internalization. Cells were then fixed and permeabilized before they were stained again with mouse anti-HA-tag primary antibodies and anti-mouse atto488-conjugated secondary antibodies.

### Counterstaining of Intracellular Compartments

The plasmids to fluorescently label the cell membrane (pYFP-Mem), the ER (pYFP-ER), mitochondria (pYFP-mito) and peroxisomes (pEGFP-Pex) were obtained from Clontech. The plasmid to label the endosomes (GFP-Rab5; Addgene plasmid # 31733) was a gift from Richard Pagano (Choudhury et al., 2002). Lysosomes were counterstained with Lamp1-GFP.

Co-localization was observed using Zeiss LSM 510 Meta system. Quantitative analysis of co-localization was carried out by calculating Pearson’s correlation coefficients using LSM 510 software.

### Confocal Laser-Scanning Microscopy

Distribution of recombinant receptors, organelle markers and immuno-fluorescent staining was analyzed by microscopy using a confocal laser scanning microscope LSM 510 Meta (Carl Zeiss, Jena, Germany) equipped with a 63× plan-apochromatic phase-contrast water-immersion objective, NA 1.4. Fluorophores were excited either with light at 458 nm, 488 nm or 514 nm from an Argon laser, at 543 nm from a HeNe laser or at 633 nm from a second HeNe laser. Emission of the fluorophores was recorded using the Meta detector. Analysis, e.g., intensity measurements, were performed by importing the images into ImageJ (Schneider et al., 2012). For plate assembly, images were digitally adjusted for brightness and contrast if necessary.

### Förster Resonance Energy Transfer (FRET) Acceptor Photobleaching

Cells were transfected with equimolar amounts of 5-HT_1A_-GFP and 5-ht_5b_-mCherry or with GFP-rab5 and mRFP-rab5 (a gift from Ari Helenius (Addgene plasmid # 14437, Vonderheit and Helenius, 2005) or with single plasmids for negative control and fixed using 2% paraformaldehyde for 20 min prior to imaging. After collecting three images in both the GFP and the mRFP channels, the mRFP in the regions of interests (ROIs) were photobleached by scanning 50 times at high laser power. After photobleaching, again three images of both channels were taken. The obtained images were averaged to calculate the GFP_before_, GFP_after,_ mRFP_before_ and mRFP_after_ values. The apparent Förster Resonance Energy Transfer (FRET) efficiency was calculated using the following formula: Apparent FRET efficiency = ((GFP_after_ − GFP_before_) × mRFP_before_) × ((GFP_after_ × mRFP_before_) − (GFP_before_ × mRFP_after_))^−1^ (van Royen et al., 2009). Image analysis was performed in ImageJ (Schneider et al., 2012).

### Fluorescence Activated Cell Sorting (FACS) Analysis

To determine to what extent 5-ht_5b_ expression affects translocation of 5-HT_1A_ to intracellular organelles, we performed QD-mediated fluorescence activated cell sorting (FACS) analysis to measure the amount of surface expression of HA-5-HT_1A_. Cells expressing HA-5-HT_1A_ and 5-ht_5b_ at equimolar ratios were compared to cells expressing HA-5-HT_1A_ transfected with the same amount of DNA together with an equimolar amount of unspecific cDNA to compensate for transcriptional effects. Cells were fixed in 2% paraformaldehyde/PBS for 15 min and labeled with excess of primary antibody against rabbit-anti-HA-tag (Santa Cruz, sc-805) and with a secondary antibody coupled to quantum dots (655 nm, Invitrogen). Avoiding cell permeabilization as well as the bulk of the QDs ensured that no intracellular proteins were labeled. Samples were run on a modified LSR II instrument from Becton and Dickinson using 405 nm excitation and a 660/40 nm emission filter.

### Quantification of cAMP Signaling

The ability of 5-HT to modulate cAMP levels in cells under pharmacological treatment was assessed with the cAMP-Glo assay (Promega) according to the manufacturer’s instructions. cAMP amounts were determined by fitting the results from triplicate experiments to the standard curve. The phosphodiesterase inhibitor IBMX (50 mM) was present during this treatment.

### Statistical Analysis

Statistical significance of the data was tested by one-way-ANOVA with Bonferroni’s post-test for multiple comparisons using GraphPad Prism 6 (GraphPad Software Inc., La Jolla, CA, USA) on a Microsoft Windows PC. A *p*-value of less than 0.05 was considered significant. Data are presented as mean ± standard error of the mean (SEM).

## Results

### The 5-ht_5b_ Receptor Shows an Unusual Intracellular Localization

**Figure 1:**
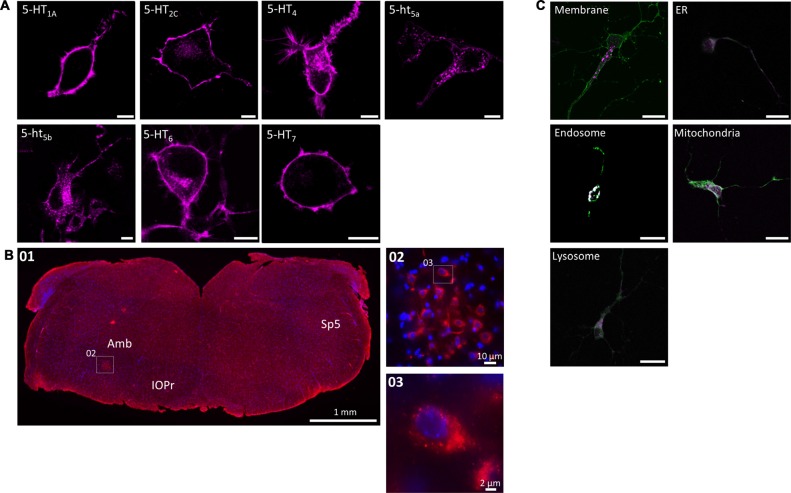
Subcellular localization of serotonin (5-HT) receptors *in vitro* and *in vivo*. **(A)** When expressed recombinantly in N1E-115 cells, 5-HT_1A/2C/4/6/7_ produce a clear membrane staining. In contrast, 5-ht_5a/5b_ produce a distinct intracellular staining with weak (5-ht_5a_) or no (5-ht_5b_) membrane staining. Artificially truncating the other 5-HT receptors (here shown exemplarily for 5-HT_7_) produces the same intracellular pattern observed for 5-ht_5a/5b_. Scale bars represent 10 μm. **(B)** The intracellular localization of 5-ht_5b_ is not an artifact of recombinant expression, but can be visualized *in vivo*. Immunohistological staining of murine brainstem (01) from hemizygous *Mecp*^+/−^ mice against 5-ht_5b_ (see “Materials and Methods” Section) reveals upon magnification (02) the same clustered intracellular localization (03) as seen in cells. Scale bars as indicated. Bregma is ~ −6.5. Abbreviations: Amb, Nucleus ambiguus; IOPr, inferior olive, principal nucleus; Sp5, spinal trigeminal tract. **(C)** Co-localization of 5-HT receptor 5-ht_5b_ (purple) with cellular compartment markers (green) in primary hippocampal neurons. Neurons between DIV 9 to DIV 11 were transfected with fluorescently labeled 5-HT receptor 5-ht_5b_ and compartment markers for membrane (GAP-43), endoplasmic reticulum (ER; recognition sequence of calreticulin), mitochondria (recognition sequence of cytochrome C-oxidase), lysosomes (Lamp-1) and endosomes (rab5). The strongest co-localization is with the endosomal marker. Scale bars in all images are 20 μm.

When expressed in N1E-115 cells, most 5-HT receptors produced a clear membrane-localized signal with minor staining of the Golgi apparatus due to trafficking (Figure 1A). With 5-HT_4_ and 5-HT_6_, membrane staining was strong, however a moderate amount of punctate clusters were visible in transfected cells. Only 5-ht_5a_ and 5-ht_5b_ showed a strong, punctate intracellular localization with little (5-ht_5a_) or no (5-ht_5b_) membrane staining at all. We have previously described that 5-ht_5b_ is retained in intracellular organelles with the most significant co-localization found with endosomes and fluorescence immunohistochemistry on mouse brainstem sections detected 5-ht_5b_ positive cells (Figure 1B01). High magnification images of this area revealed a clustered sub-cellular localization for 5-ht_5b_ in these cells (Figures 1B02,03). 5-ht_5b_ shows massive dysregulation in a mouse model of RTT (Vogelgesang et al., 2017). To follow up on the previously published results, we tested the sub-cellular localization of 5-ht_5b_ in primary hippocampal neurons (Figure 1C) and could verify the results from N1E-115 cells with the highest co-localization of 5-ht_5b_ with endosomal marker rab5 (0.867 ± 0.016), while markers for membrane (0.035 ± 0.007), mitochondria (0.538 ± 0.06), ER (0.273 ± 0.17) and lysosome (0.471 ± 0.11) showed little overlap.

### N-Terminal Glycosylation of 5-ht_5_ Receptors Controls Intracellular Localization

**Figure 2:**
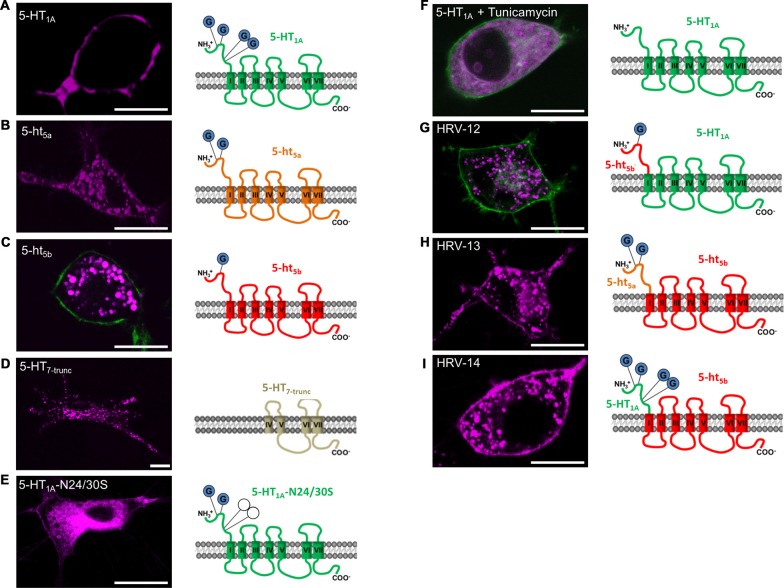
Effect of N-terminal glycosylation on membrane trafficking of 5-HT receptors. For all experiments, N1E-115 cells were transiently transfected with the same amount of receptor corresponding deoxyribonucleic acid (cDNA). All receptor constructs are shown in purple. If the outline of the cell was not easily recognized, a membrane label (GAP-43) was co-transfected (green).** (A)** 5-HT_1A_ possesses four glycosylation sites on the N-terminus and displays specific membrane localization. **(B)** 5-ht_5a_ possesses only two glycosylation sites. In comparison to the other 5-HT receptors, 5-ht_5a_ shows weak membrane staining, but pronounced staining of endosomal compartments. **(C)** 5-ht_5b_ possesses only one glycosylation site and membrane localization of the receptor is not detectable. **(D)** Artificially truncated 5-HT receptor 5-HT7 (5-HT7-trunc) comprises the trans-membrane domains (TMDs) IV to VII and shows strong intracellular clustering with no discernable membrane staining. **(E)** Site-directed mutagenesis of Asparagine (N) to Serine (S) at positions 24 and 30 in 5-HT_1A_-N24/30S removes two of four glycosylation sites and greatly reduces membrane trafficking of the mutated receptor. **(F)** Tunicamycin blocks the formation of protein N-glycosidic linkages by inhibiting the transfer of N-acetylglycosamine-1-phosphate to dilichol mono-phosphate. Application of Tunicamycin (1 μg/ml) to cells expressing 5-HT_1A_ removes the receptor from the cell membrane. **(G)** Replacing the N-terminal domain of 5-HT_1A_ containing 4 glycosylation sites with the N-terminal domain of 5-ht_5b_ with 1 glycosylation site completely abolishes membrane localization of the hybrid receptor and produces a clustered intracellular staining. **(H)** Replacing the N-terminal domain of 5-ht_5b_ containing 1 glycosylation site with the N-terminal domain of 5-ht_5a_ with 2 glycosylation sites produces a clustered intracellular staining and produces moderate membrane localization. **(I)** Replacing the N-terminal domain of 5-ht_5b_ containing 1 glycosylation site with the N-terminal domain of 5-HT_1A_ with 4 glycosylation sites does not prevent intracellular localization, but leads to significant membrane localization of the hybrid receptor. Scale bars in all images are 10 μm.

Based on literature and the truncation experiments, the most likely mechanism responsible for sorting proteins between membrane and intracellular organelles other than distinct localization signals is glycosylation of the N-terminus (Vagin et al., 2009; Guangyu, 2015). An influence of glycosylation on the trafficking of 5-ht_5a_ was also shown previously (Dutton et al., 2008), with two glycosylation sites identified at N6 and N21 (Figure 2B). Incidentally, the intracellular clusters of 5-ht_5a_ also co-localize strongest with endosomal markers (data not shown). Analyzing the protein sequences of 5-HT receptors, we found that strictly plasma membrane localized 5-HT_1A_ receptor possess four N-terminal glycosylation sites (Figure 2A) at positions N10, N11, N24 and N30, while 5-ht_5b_ receptors (Figure 2C) possess only one glycosylation site at position N5. As 5-ht_5b_ can be detected in Western blots of murine brain both as a full length as well as a truncated protein (Vogelgesang et al., 2017), we tested if the truncation causes intracellular localization. An artificially truncated 5-HT_7-trunc_ produced a similar staining with completely absent membrane localization and fluorescence concentrated in intracellular organelles (Figure 2D). However, 5-HT_7-trunc_ co-localized strongest with the lysosomal marker (data not shown). Thus, the artificial truncation led to a defective receptor that either folds incorrectly or is otherwise marked for degradation and is sent to the lysosome.

In contrast, the number of glycosylation sites corresponds well with the strength of membrane expression with no discernable membrane staining in case of 5-ht_5b_ (Figure 2C), weak staining for 5-ht_5a_ (Figure 2B) and strong staining for 5-HT_1A_ (Figure 2A).

Successive removal of glycosylation sites in 5-HT_1A_ by site-directed mutagenesis led to increasing loss of surface expression. While removing one glycosylation site had no visible effect (data not shown), removal of two glycosylation sites produced only weak surface expression and strong endosomal accumulation of the receptor (Figure 2E). Removal of three or more glycosylation sites led to a total loss of surface expression (data not shown). Complete, yet unspecific de-glycosylation of 5-HT_1A_ receptors with 1 μM Tunicamycin (Figure 2F) blocked cell surface localization of the receptor indicating a glycosylation dependence of its trafficking.

Hybrid receptor constructs composed of the extracellular N-terminus of 5-HT_1A_ or 5-ht_5a_ receptors and the 5-ht_5b_ receptor (Figures 2H,I) reconstitute 5-ht_5b_ receptor localization on the cell membrane with staining intensity depending on the strength of the glycosylation signal. In contrast, fusion of the N-terminus of 5-ht_5b_ to 5-HT_1A_ abolishes membrane localization (Figure 2G). As it had already been confirmed for 5-ht_5a_ (Dutton et al., 2008), we concluded that N-terminal glycosylation also determines the membrane trafficking of 5-ht_5b_.

### 5-ht_5b_ Specifically Interacts with 5-HT_1A_

Knowing that many GPCRs (Gurevich and Gurevich, 2008; Schwenk et al., 2010) and some 5-HT receptors in particular (Pellissier et al., 2011; Renner et al., 2012) form homo- or heterodimers, we wanted to know whether co-expression with other 5-HT receptors could lead to a stronger surface expression of 5-ht_5b_.

**Figure 3:**
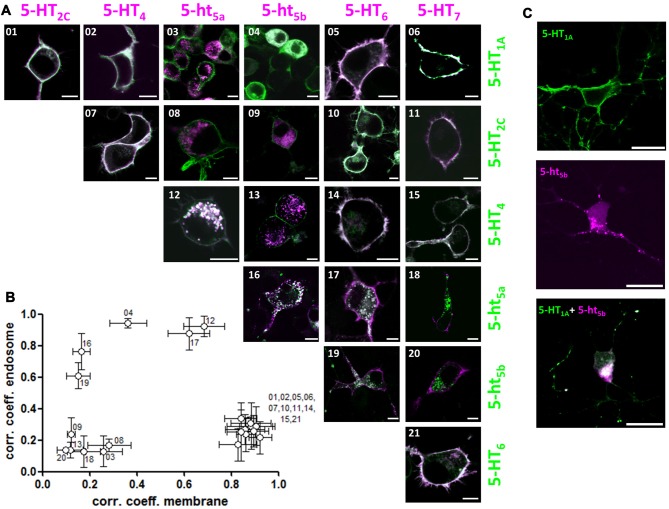
5-ht_5b_ specifically interacts with 5-HT_1A_ to change the latter’s subcellular localization. **(A)** We transfected a representative member of each 5-HT receptor subgroup tagged with mCherry against the same set of 5-HT receptors tagged with GFP. Each image of the matrix shows N1E-115 cells co-expressing two 5-HT receptors. The images are pseudo-colored purple for the receptor indicated horizontally and pseudo-colored green for the receptor indicated vertically. Co-localization is shown in yellow. 5-ht_5b_ specifically interacts with 5-HT_1A_ (04) with the normally strictly membrane-bound 5-HT_1A_ receptors partly retained intracellularly. In addition, 5-ht_5a_ seems to interact with 5-HT_4_ (12) and 5-HT_6_ seems to weakly interact with 5-ht_5a_ (17) and 5-ht_5b_ (19), although 5-HT_6_’s surface expression remains strong. No other 5-HT receptors seem to interact with each other in a way that changes subcellular localization. Scale bars in all images are 10 μm. **(B)** For quantification, we separately calculated Pearson’s correlation coefficients for the fluorescence signals at the membrane and at intracellular organelles by manually applying regions of interest (ROI). Plotting the organelle correlation coefficient over the membrane correlation coefficient for each receptor pair resulted in three populations of receptors. One population displays a high correlation at the membrane, but not at intracellular organelles. The second population displays correlation in neither compartment while the third population displays high correlation at intracellular compartments. **(C)** To verify interaction of 5-HT_1A_ and 5-ht_5b_, we transfected primary hippocampal neurons with expression constructs of both receptors. 5-HT_1A_ (green) localizes to the cell membrane, while 5-ht_5b_ (purple) localizes to intracellular organelles, while co-expression of both receptors shows intracellular co-localization with no discernable membrane staining by 5-HT_1A_. Scale bars in all images are 20 μm.

We co-expressed a representative member of each 5-HT receptor subgroup tagged with mCherry against the same set of 5-HT receptors now tagged with GFP (Figure 3A). However, we excluded 5-HT_3_R in this matrix because it is a multimeric ion channel (Uetz et al., 1994; Boyd et al., 2002). Quantification of the correlation coefficients revealed three separate populations (Figure 3B). Here, one population corresponds to the receptor pairs that co-localize to the plasma membrane while the other corresponds to receptor pairs that remain separated between membrane and intracellular organelles. The third population was comprised of the receptor pairs that showed possible interaction. Of this group, the receptor pair of 5-HT_1A_ and 5-ht_5b_ displayed the most unusual behavior, with 5-ht_5b_ not showing enhanced surface expression, but instead retaining 5-HT_1A_ in the endosomal compartments with select specificity (Figure 3A04). This change of 5-HT_1A_’s sub-cellular localization could also be reproduced in primary neurons (Figure 3C).

After observing the retention of 5-HT_1A_ on intracellular organelles by 5-ht_5b_, we looked for the mechanism. With GPCRs known to form oligomers, direct interaction was an obvious answer, but overloading of the transport pathway was also possible. To differentiate between both mechanisms, we performed a series of experiments while always using equal amounts of transfected DNA.

An early indicator for interaction was the specificity of interaction. The change of subcellular localization only happens between 5-HT_1A_ and 5-ht_5b_, but not between 5-HT_1A_ and any other receptor or 5-ht_5b_ and any other receptor.

**Figure 4:**
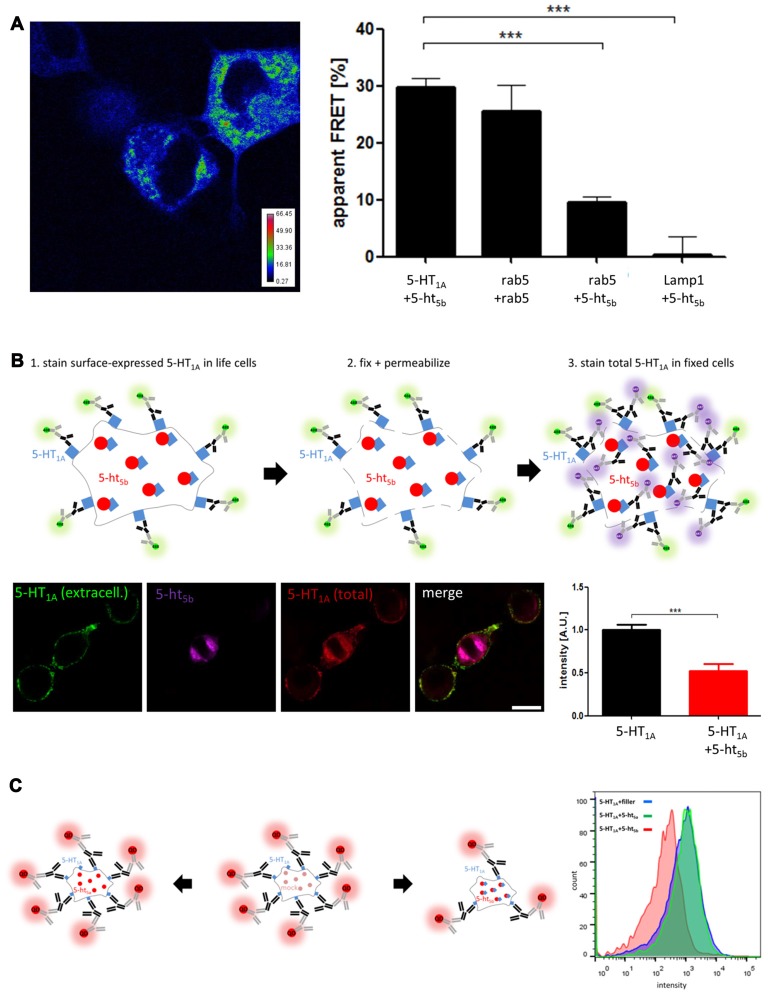
Analysis of receptor interaction. **(A)** Acceptor-photobleaching Förster Resonance Energy Transfer (abFRET) microscopy was used to determine the extent of protein:protein-interaction. Protein pairs of 5-HT_1A_:5-ht_5b_, rab5:rab5, 5-ht_5b_:rab5 and 5-ht_5b_:Lamp1 were co-expressed in N1E-115 cells at equimolar concentrations and the apparent FRET efficiency (F_app_) was measured. The color-coded image shows an exemplary experiment of 5-HT_1A_:5-ht_5b_, while the bar diagram shows the apparent FRET efficiencies of 5-HT_1A_:5-ht_5b_ (29.74 ± 1.6), rab5:rab5 (25.58 ± 4.6), 5-ht_5b_:rab5 (9.56 ± 1.1) and 5-ht_5b_:Lamp1 (0.02 ± 0.7). Asterisks indicate significance (***p ≤ 0.001; one-way-ANOVA with Bonferroni’s post-test for multiple comparisons). **(B)** Immunohistochemical analysis of distribution and equal expression levels. Non-permeabilized cells were stained for 5-HT_1A_ (green), then fixed and permeabilized and stained again for 5-HT_1A_ (purple). 5-ht_5b_ is co-expressed in some cells as a fusion protein with a fluorescent tag (red). The fluorescence intensity of the first 5-HT_1A_ staining was then measured in cells expressing 5-HT_1A_ and in cells expressing 5-HT_1A_ and 5-ht_5b_. The cartoon shows the experimental procedure. The bar diagram shows the mean fluorescence intensities of 5-HT_1A_ reactivity in cells expressing only 5-HT_1A_ and in cells expressing 5-HT_1A_ (1.0 ± 0.06) and 5-ht_5b_ (0.52 ± 0.08) (***p ≤ 0.001; unpaired *t*-test). **(C)** The effect of 5-HT_1A_:5-ht_5b_ co-expression on 5-HT_1A_ surface localization was quantified by fluorescence activated cell sorting (FACS) analysis of N1E-115 cells expressing HA-5-HT_1A_ alone or together with 5-ht_5b_. Expressing 5-HT_1A_ together with an equal amount of filler DNA was used to determine 5-HT_1A_ surface expression under control conditions (blue). Co-expression of 5-HT_1A_ with an equal amount of non-interacting 5-ht_5a_ did not change the degree of surface expression (green). Co-expression of 5-HT_1A_ with an equal amount of 5-ht_5b_ decreased the cell surface expression of 5-HT_1A_ by 48.8% ± 17.4%. The cartoon shows the experimental procedure. Labeling was directed at HA-tag on 5-HT_1A_ with quantum dot-coupled secondary antibodies. To avoid skewing results by transcription level effects in the control measurement, an unspecific filler plasmid (mock) was co-transfected in place of the second receptor to maintain the same total amount of transfected plasmid.

First, we tested direct interaction using acceptor-photobleaching FRET (abFRET) microscopy (Figure 4A) using GFP- and mRFP-labeled constructs (see Materials for details). Apparent FRET efficiency (F_app_) of the two 5-HT receptors was 29.74% ± 1.6, which was even slightly higher than two rab5’s expressed together (25.58% ± 4.6). Here, a strong FRET signal is expected as the proteins localize to the same organelles and are therefore in close proximity. Testing unspecific interaction by co-expressing 5-ht_5b_ with rab5 showed a significantly lower F_app_ of 9.56% ± 1.1. Here, some FRET might be expected, as 5-ht_5b_ co-localizes with endosomes (Figure 1C), but the FRET signal was significantly smaller compared to the 5-HT_1A_:5-ht_5b_ signal. The protein pair of 5-ht_5b_ and Lamp1 produced a very low F_app_ of 0.02% ± 0.7, as the proteins are on separate organelles (Vogelgesang et al., 2017) and only interact very little.

Then, to quantify the surface expression of 5-HT_1A_ in the absence and in the presence of 5-ht_5b_, we used fluorescence microcopy (Figure 4B). First, we stained only surface-expressed 5-HT_1A_ (green) in live cells, as antibodies cannot penetrate through the membrane. Then we fixed and permeabilized the cells and stained 5-HT_1A_ again with different primary and secondary antibodies, this time staining both surface-expressed as well as internal receptors (purple). Cells co-expressing 5-ht_5b_ were identified by its inherent mCherry-fusion fluorescent signal (red). We quantified the fluorescence intensity of surface-expressed 5-HT_1A_ (green) in cells with and without 5-ht_5b_ signal and found a reduction of 5-HT_1A_ surface expression by 52.3% ± 8.05 in cells co-expressing 5-ht_5b_.

To quantify 5-HT_1A_ in a larger amount of cells, we used FACS (Figure 4C). Cells expressing 5-HT_1A_ together with an equal amount of filler DNA was used to determine 5-HT_1A_ surface expression under control conditions. Co-expression of 5-HT_1A_ with an equal amount of 5-ht_5a_ did not change the degree of surface expression. This indicates that the mere presence of another plasmid or receptor did not affect the membrane localization of 5-HT_1A_. However, the surface expression of 5-HT_1A_ was reduced by 48.8% ± 17.4% when it was expressed with an equal amount of 5-ht_5b_ (Figure 4C). The reduction of surface-expressed 5-HT_1A_ was consistent with the reduction seen with immunohistochemistry (Figure 4B).

While the translocation of 5-HT_1A_ to a different sub-cellular localization was obvious (Figure 3A04), the amount of surface availability could also be affected by different expression levels caused through co-transfection. As expression of all receptor constructs is CMV driven and all construct have roughly the same size, we regard this as unlikely. Nevertheless, we analyzed whether expression levels of 5-HT_1A_ were affected by co-transfection by comparing the fluorescence intensity of 5-HT_1A_-GFP in cells expressing equal amounts of 5-HT_1A_ and a filler DNA or cells expressing 5-HT_1A_ and 5-ht_5b_. Although expression levels of both receptors fluctuated in individual cells, we found no significant overall differences (Supplementary Figure S1) that could explain the 2-fold reduction in surface expression seen above.

With direct interaction being the most likely mechanism, we cannot rule out indirect interaction. It has been shown previously that transport of 5-HT_1A_ in neurons is controlled by co-factor Yif1b (Carrel et al., 2008), but the involvement of adaptor proteins will be the topic of further studies.

While the change in subcellular localization was most pronounced for 5-HT_1A_ by 5-ht_5b_, for completeness we would like to mention additional possible interactions that were revealed by plotting the correlation coefficients (Figure 3B). We found that 5-ht_5a_ co-localized with 5-HT_4_ (Figure 3A12) and that both 5-ht_5_ receptors showed moderate co-localization with 5-HT_6_ (Figures 3A17 and 3A19). Although intracellular aggregation became more pronounced upon co-expression, both 5-HT_4_ and 5-HT_6_ showed already minor intracellular clusters when expressed alone (Figure 1A). In these cases, overloaded trafficking is a more likely mechanism, but analysis is more complicated and will be done at a later time.

### Co-Expression of Serotonin Receptors in the Mouse Brain

**Figure 5:**
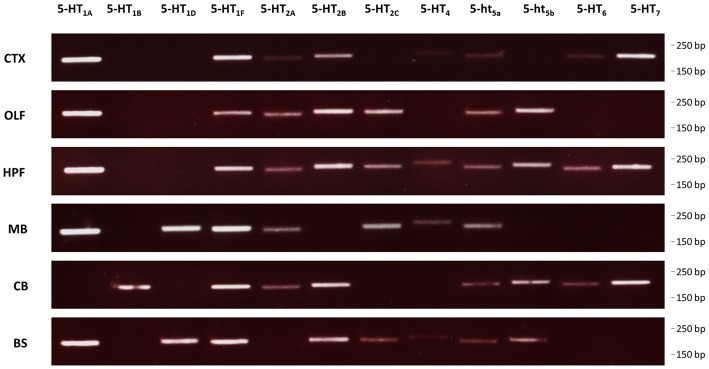
Expression of 5-HT receptors in the mouse brain. RT-PCR was used to detect expression of all 5-HT receptors in select brain regions of mice. CTX, cortex; OLF, bulbus olfactorius; HFP, hippocampal formation; MB, midbrain; CB, cerebellum; BS, brainstem.

We used RT-PCR to determine the expression of all receptors in different regions of the brain (Figure 5). RT-PCR confirmed wide-spread 5-HT receptor expression in the murine brain with multiple receptors being expressed in basically every region analyzed. Among these, the hippocampal formation showed the largest diversity of all regions analyzed. Specifically, for 5-HT_1A_ and 5-ht_5b_, we found regional co-expression in the bulbus olfactorius, the hippocampal formation and the brainstem.

### Identification of Interaction Domains

**Figure 6:**
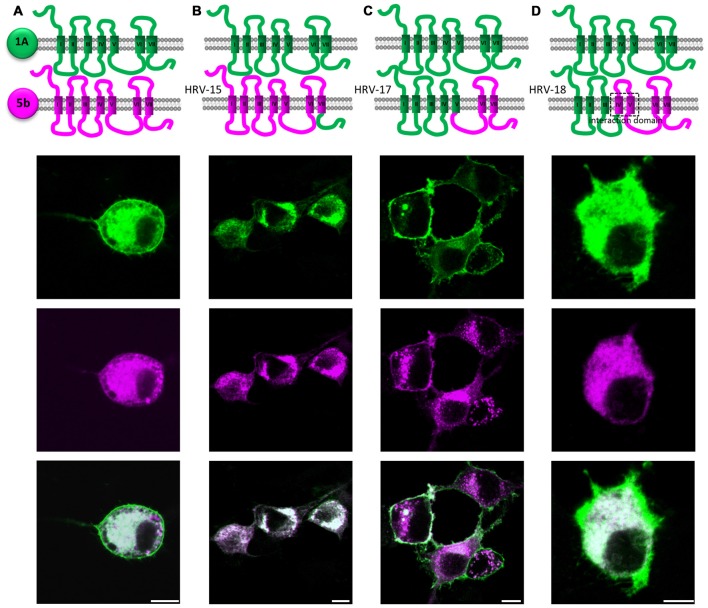
Identification of possible interaction domains between 5-HT_1A_ (green) and 5-ht_5b_ (purple). N1E-115 cells were transiently transfected with two hybrid receptor variants labeled with eGFP or mCherry. **(A)** Co-expression of full length 5-HT_1A_ and full length 5-ht_5b_ lead to strong internalization of 5-HT_1A_. **(B)** Replacing the C-terminal domain of 5-ht_5b_ with the C-terminal domain of 5-HT_1A_ (HRV-15) did not change the amount of interaction. **(C)** Expressing 5-HT_1A_ together with a hybrid 5-HT_1A_ where the 3rd intracellular loop and the last two TMDs are from 5-ht_5b_ (HRV-17), the interaction is greatly reduced or even abolished. **(D)** Expressing 5-HT_1A_ together with a hybrid receptor composed of the first three TMDs of 5-HT_1A_ and the last four TMDs of 5-ht_5b_ (HRV-18) show interaction as the two full length receptors.

Having prepared some hybrid receptors for the study of glycosylation and trafficking, we continued this strategy to try and define domains which would explain the specific interaction between 5-HT_1A_ and 5-ht_5b_ (Figure 6A).

Replacing the PDZ-like C-terminal domain of 5-ht_5b_ with that of 5-HT_1A_ had no influence on subcellular localization or interaction (HRV-15, Figure 6B).

A hybrid receptor (HRV-17) consisting of 5-HT_1A_ (from start up to TMD V) and 5-ht_5b_ (intracellular loop 3 (ICL_3_) and onwards) can interact with wild type 5-HT_1A_, although not as strong as seen between both wild-type receptors. This indicated that ICL_3_ contains at least parts of the interaction domain (Figure 6C).

Exchanging TMD IV and V of 5-HT_1A_ in the previous hybrid clone by TMD IV and TMD V of 5-ht_5b_, thus creating HRV-18, fully restored the interaction to levels seen for the wild-type receptors (Figure 6D).

### Physiological Consequences of 5-ht_5b_ Expression

The strong and far-ranging expression of 5-ht_5b_ (Figure 5) indicates some physiological significance. However, given its intracellular expression, 5-ht_5b_ differs from classical GPCRs. With at best sparse surface expression, 5-ht_5b_ seems to contribute little to intracellular signaling. However, a more likely function of 5-ht_5b_ could be the modulation of its interaction partner by affecting its surface availability.

**Figure 7:**
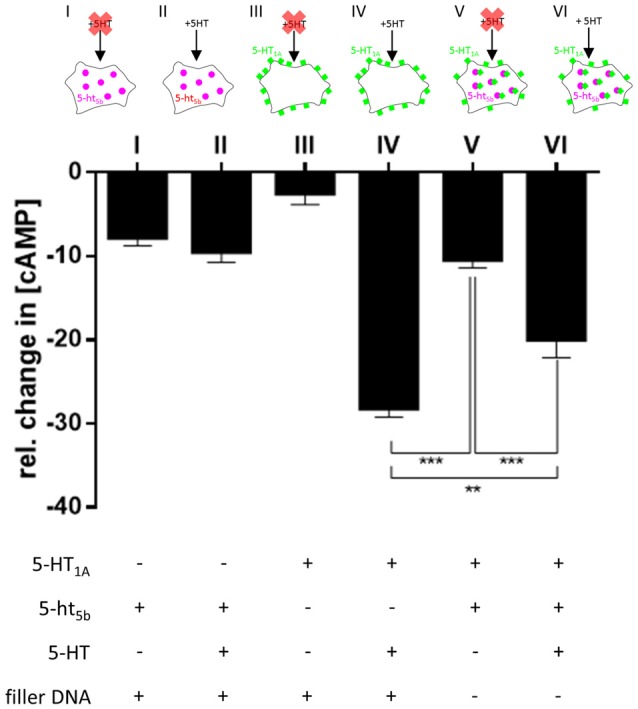
Physiological consequences of 5-HT_1A_:5-ht_5b_ co-expression. To test whether translocation of 5-HT_1A_ by 5-ht_5b_ effects cyclic adenosine monophosphate (cAMP) signaling, we measured the cellular cAMP level under various conditions. To measure the decrease of cAMP, 50 nM Forskolin was added to all cells before the start the experiment to increase cAMP levels. Mock-transfected cells treated with Forskolin and stimulated with 1 μM 5-HT served as control and were set to 100%. In all experiments, cells were transfected with an equimolar amount of DNA. To keep the amount of DNA the same in experiments with only one receptor, a filler plasmid was co-transfected. Expression of 5-ht_5b_ without stimulation with 5-HT decreased cAMP by −7.75% ± 1.012 (I), while addition of 5-HT did not enhance the reaction (−9.46% ± 1.296; II). Expressing 5-HT_1A_ in cells had no effect on cAMP levels without stimulation (−2.49% ± 1.366; III), but adding 5-HT decreased cAMP by −28.12% ± 1.096 (IV). Co-expression of 5-HT_1A_ and 5-ht_5b_ together without stimulation by 5-HT lead to a drop in cAMP by −10.37% ± 1.029 (V). Adding 5-HT to co-expressing cells dropped the cAMP level by −19.91% ± 2.219 (VI). Asterisks indicate significance (**p ≤ 0.01; ***p ≤ 0.001; one-way-ANOVA with Bonferroni’s post-test for multiple comparisons).

The strictly intracellular 5-ht_5b_ did not respond to stimulation by 5-HT (−9.46% ± 1.296, Figure 7II), however, the mere presence of 5-ht_5b_ decreased the cAMP level, indicating some constitutive activity of 5-ht_5b_ (−7.75% ± 1.012, Figure 7I). For 5-HT_1A_ no constitutive activity was measurable (−2.49% ± 1.366, Figure 7III), while application of 5-HT decreased cAMP (−28.12% ± 1.096, Figure 7IV). Cells transfected with both receptors showed reduced cAMP levels (−10.37% ± 1.029, Figure 7V). Application of 5-HT to cells co-expressing both receptors produced a reduction of cAMP (−19.91% ± 2.219, Figure 7VI), as activation of 5-HT_1A_ decreased cAMP despite the reduced baseline cAMP level due to 5-ht_5b_’s constitutive activity. The reduction of cAMP was smaller compared to cells expressing 5-HT_1A_ alone, yet significant (*p* = 0.002). This clearly showed that co-expression of 5-ht_5b_ can affect the signaling behavior of other 5-HT receptors.

## Discussion

In this study we described the behavior of 5-HT receptor 5-ht_5b_, which manifests in intracellular localization to endosomal compartments and specific interaction with another select member of the 5-HT receptor family.

5-ht_5b_ localizes to endosomes (Figure 1C), which was also seen for 5-ht_5a_ (data not shown) and therefore might present a defining feature of this sub-group of 5-HT receptors. It might also indicate a physiological significance, as other truncated receptors are retained mainly in the ER (Karpa et al., 2000; Leung et al., 2007; Gonzalez et al., 2011). It is known that many GPCRs naturally show constitutive activity or that constitutively active mutants exist (Tao, 2008). GPCRs were even described to signal from endosomal vesicles after internalization (Irannejad et al., 2013). Internal signaling might be indicated by the fact that we could measure a minor decrease of cAMP upon expression of 5-ht_5b_ without application of a ligand (Figure 7).

In the literature, several mechanisms, including glycosylation (Dutton et al., 2008), TMD size (Sharpe et al., 2010) or N-terminal signal peptides (Jahnsen and Uhlen, 2012) are discussed to control receptor trafficking to the membrane. The unusual intracellular localization of 5-ht_5a_ and 5-ht_5b_ seems to be caused in large part by glycosylation of the N-terminus as artificially increasing the number of glycosylation sites of 5-ht_5b_ increases its surface localization (Figures 2H,I). This form of glycosylation was also found to control membrane localization of 5-ht_5a_ (Dutton et al., 2008).

Formation of hetero-oligomers has been described for many GPCRs (Schwenk et al., 2010) including 5-HT receptors (Pellissier et al., 2011; Renner et al., 2012), although their interaction is subtle and its physiological significance remains to be shown. Here, 5-ht_5b_‘s affinity to 5-HT_1A_ was so strong that it changes the subcellular localization of 5-HT_1A_. As both 5-HT receptors were found co-expressed in the brain (Figure 5), it is feasible to assume a physiological role for this specific interaction. The interaction mapping is still only rudimentary, but efforts to identify the binding interfaces of class A GPCRs have been ongoing for years, with most studies leaning towards TMD IV and TMD V as dimerization interface (González-Maeso et al., 2008; Johnston et al., 2011; Hu et al., 2012). These were also identified here as a possible location for the interaction interface. With the strong interaction between 5-HT_1A_ and 5-ht_5b_ and an easy read-out through translocation, further studies to fine-map the interaction surface might profit.

The mostly intracellular 5-ht_5b_ receptor probably does not relay signals in the conventional way, but by retaining 5-HT_1A_ in intracellular compartments, 5-ht_5b_ reduces its surface expression significantly, thus effectively reducing 5-HT_1A_’s contributions to cellular signaling (Figures 4, 7). In alternate modes of action, 5-ht_5b_ might modify intracellular signaling by acting on the cAMP level, either through constitutive activity and/or by scavenging G-proteins which would no longer be available for other signal cascades.

It is difficult to define a role for the 5-HT_1A_:5-ht_5b_ interaction in RTT, as the syndrome displays very complex genetic imbalances and many different and sometimes opposing therapies seem to improve symptoms in RTT. Within the serotonergic system alone, two previous observations in animal models of RTT might be linked to 5-HT_1A_:5-ht_5b_. Application of 5-HT_1A_ agonists was described to have beneficial effects on the respiratory phenotype in mice (Abdala et al., 2010) and might be linked to the reduction of surface-expressed 5-HT_1A_ by 5-ht_5b_. In contrast, treatment with 5-HT_7_ agonists was reported to improve behavior (De Filippis et al., 2014, 2015a) and might counteract the constitutive down-regulation of cAMP by 5-ht_5b_.

However, we would like to advise some caution interpreting these results. Here, we transfected recombinant cDNA at equimolar concentrations into cell lines and measured the effect of receptor activation on cAMP. *In vivo*, the situation is most likely much more complicated, as receptor expression levels may not be equal, co-factors might be present and signaling cascades are more complex. For example, we did not measure effects on CNG ion channels or on small GTPases. Of interest regarding the physiological function of 5-ht_5b_ is the fact that 5-HT_1A_ also becomes internalized upon activation, after which it seems to activate the Erk-1/2 signaling pathway (Renner et al., 2012). This is especially interesting as 5-ht_5b_ initially came to our attention when analyzing a mouse model of RTT (Vogelgesang et al., 2017) and modulation of small RhoGTPase activity was shown to have beneficial effects on RTT (De Filippis et al., 2012, 2015b).

## Author Contributions

SN, GJB, SV, TM and MN performed experiments. SN and MN wrote the manuscript.

## Conflict of Interest Statement

The authors declare that the research was conducted in the absence of any commercial or financial relationships that could be construed as a potential conflict of interest.
