# Supplementary figures and images for: The Serotonin Receptor Subtype 5b Specifically Interacts with Serotonin Receptor Subtype 1A
Source: Front Mol Neurosci. 2017 Sep 21;10:299. doi: 10.3389/fnmol.2017.00299 (PMC5613149; doi:10.3389/fnmol.2017.00299)

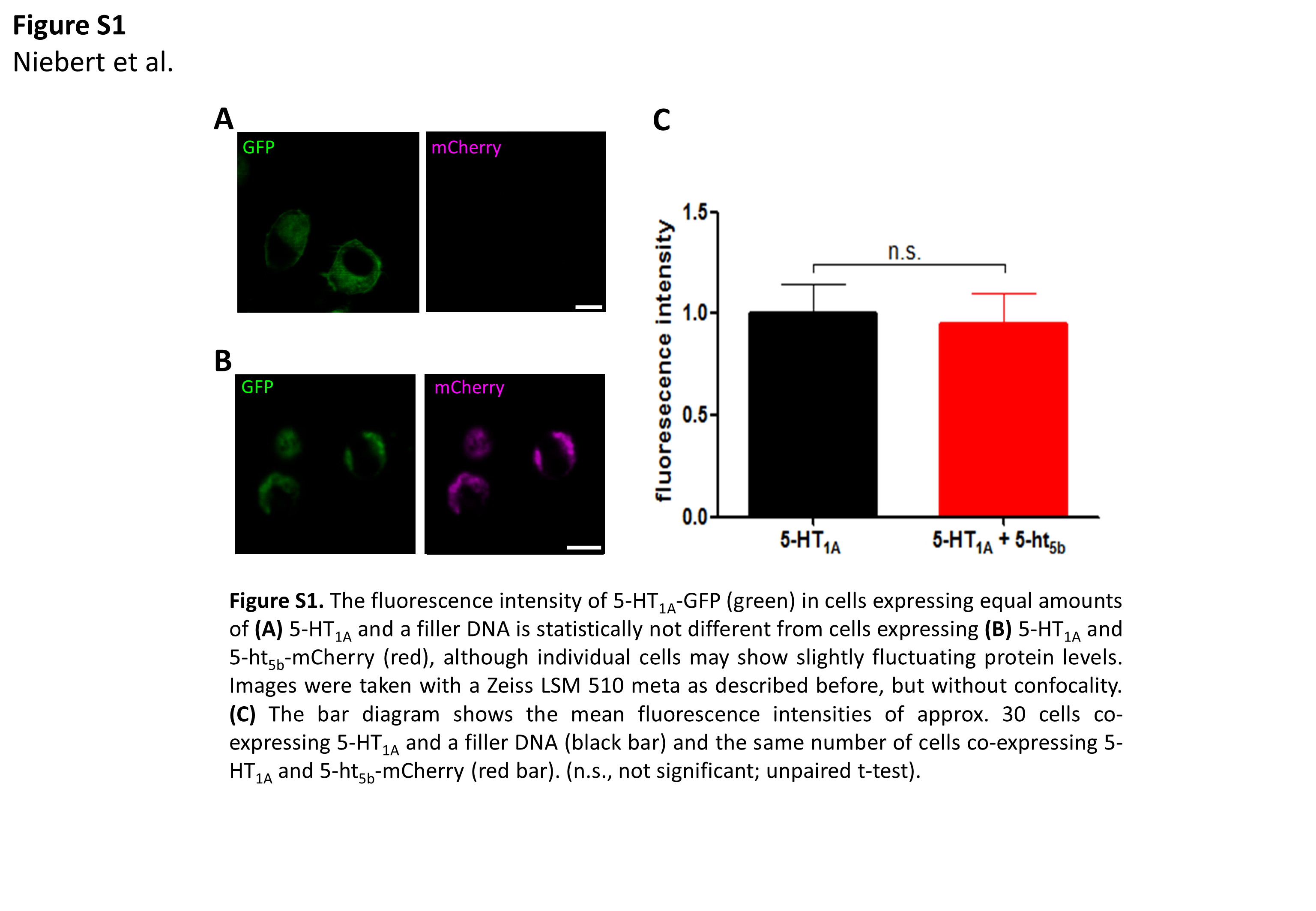

Supplement: Supplementary file 1 [file Image_1.tif]
